# Nur77 Prevents Osteoporosis by Inhibiting the NF‐κB Signalling Pathway and Osteoclast Differentiation

**DOI:** 10.1111/jcmm.17238

**Published:** 2022-02-19

**Authors:** Huanlian Tian, Feng Chen, Yingfang Wang, Yixuan Liu, Guojing Ma, Yuhong Zhao, Yanan Ma, Tingting Tian, Ruze Ma, Yang Yu, Difei Wang

**Affiliations:** ^1^ Department of Gerontology and Geriatrics Shengjing Hospital of China Medical University Shenyang Liaoning China; ^2^ Department of Health Statistics School of Public Health China Medical University Shenyang Liaoning China; ^3^ Department of General Practice Medicine Nanyang Centre Hospital Nanyang Henan China; ^4^ Department of Clinical Epidemiology Shengjing Hospital of China Medical University Shenyang Liaoning China; ^5^ Institute of Health Sciences China Medical University Shenyang Liaoning China

**Keywords:** inflammation, NF‐κB, Nur77, osteoclast, osteoporosis

## Abstract

Inflammation is a major risk factor for osteoporosis, and reducing inflammatory levels is important for the prevention of osteoporosis. Although nuclear receptor 77 (Nur77) protects against inflammation in a variety of diseases, its role in osteoporosis is unknown. Therefore, the main purpose of this study was to investigate the osteoprotective and anti‐inflammatory effects of Nur77. The microCT and haematoxylin and eosin staining results indicated that knockout of Nur77 accelerated femoral bone loss in mice. The enzyme‐linked immunosorbent assay (ELISA) results showed that knockout of Nur77 increased the serum levels of hsCRP and IL‐6. The expression levels of NF‐κB, IL‐6, TNF‐α and osteoclastogenesis factors (TRAP, NFATC1, Car2, Ctsk) in the femurs of Nur77 knockout mice were increased significantly. Furthermore, *in vitro*, shNur77 promoted the differentiation of RAW264.7 cells into osteoclasts by activating NF‐κB, which was confirmed by PDTC treatment. Mechanistically, Nur77 inhibited osteoclast differentiation by inducing IκB‐α and suppressing IKK‐β. In RAW264.7 cells, overexpression of Nur77 alleviated inflammation induced by siIκB‐α, while siIKK‐β alleviated inflammation induced by shNur77. Consistent with the *in vivo* studies, we found that compared with control group, older adults with high serum hsCRP levels were more likely to suffer from osteoporosis (OR = 1.76, *p* < 0.001). Our data suggest that Nur77 suppresses osteoclast differentiation by inhibiting the NF‐κB signalling pathway, strongly supporting the notion that Nur77 has the potential to prevent and treat osteoporosis.

## 1 INTRODUCTION

Osteoporosis is a metabolic systemic bone disease that typically manifests as decreased bone mass and increased bone fragility.^1^ With the ageing of the worldwide population, the social and economic burdens of osteoporosis remain steady. According to a previous study, approximately 15,000 osteoporotic fractures occur in the United States every year,^2^ which not only severely affects patients’ quality of life but also imposes heavy burdens on medical and health systems. The currently known risk factors for osteoporosis include age,^3^ vitamin D and calcium deficiencies,^4^ insufficient exercise,^5^ smoking,^6^ drinking,^7^ oestrogen withdrawal^8^ and inflammation,^9^, ^10^, ^11^, ^12^ and activation of the inflammatory response has attracted substantial attention, especially in older individuals.^1^

The bone organ undergoes constant remodelling that is finely orchestrated by osteoclasts and osteoblasts. During the process of bone metabolism, osteoclasts are mainly responsible for the degradation of bone matrix (bone resorption), and osteoblasts mainly synthesize new bone matrices (bone formation). However, inflammatory factors trigger osteoporosis mainly by promoting the differentiation of osteoclasts,^13^, ^14^, ^15^ which results in excessive bone resorption. The currently known inflammatory factors related to osteoporosis include NF‐κB, IL‐6, IL‐11, IL‐15, IL‐17, TNF‐α and TGF‐β,^16^, ^17^, ^18^, ^19^, ^20^, ^21^ which mainly promote the secretion of RANKL to increase osteoclast differentiation^22^; some of these factors also promote the expression of M‐CSF.^23^ However, the mechanism regulating the inflammatory response during osteoclast differentiation requires further study.

Orphan nuclear receptor 77 (Nur77) is a pivotal anti‐inflammatory factor that belongs to the NR4A subfamily and encodes *NR4A1*.^24^ Previous studies have confirmed that Nur77 vitally regulates ageing‐related processes and diseases, including apoptosis, cardiovascular system diseases, metabolism^25^, ^26^ and neurodegeneration,^27^ which share common risk factors with osteoporosis. However, to date, only a few studies have investigated the relationship between Nur77 and osteoporosis. Regarding the process of osteoclast differentiation, reliable reports on whether Nur77 can inhibit the inflammatory response are lacking.

Therefore, our study mainly explores the mechanism of Nur77 prevents osteoporosis *in vivo* and *in vitro*. We herein used Nur77 knockout mice to study the effect of Nur77 on osteoporosis and used RAW264.7 cells to study the mechanism by which Nur77 regulates osteoclast differentiation.

## 2 MATERIALS AND METHODS

### 2.1 Animals and treatments

Nur77 knockout mice on a C57BL/6J background were purchased from Jackson Laboratory (Bar Harbor, Massachusetts), and littermate wild‐type mice served as a control; 10 male mice were included in each group. All mice were housed in individually ventilated cages (IVC) in the Animal Department of China Medical University at a constant room temperature of 26°C and a humidity of 65% under a 12 h/12 h day/night cycle (daylight hours ranged from 6:00 to 18:00). All mice had ad libitum access to food and water. At the age of 8 months, the mice were anaesthetized with sodium pentobarbital, and their hearts were perfused with 1× PBS. Thereafter, the left and right femurs were collected. The left femurs were fixed in 4% paraformaldehyde, 5 samples were used for microCT detection, and 5 were subjected to pathological sectioning. The right femurs were separated, quickly frozen in liquid nitrogen and stored at −80°C, 5 femur samples were used for protein analyses, and another 5 were used for RNA extraction. All experiments were approved by the Ethics Committee of the Animal Department of China Medical University (Ethics approval project identification code: CMU2019276, Approval date: 2019‐11‐18).

### 2.2 Body weight and body composition analysis

The body weights and body compositions of the mice were measured before sampling, and the body composition was determined using a Bruker Minispec LF50. Finally, the fat mass and lean mass were used to display the body composition.

### 2.3 Serum analysis

Blood samples were collected from the fundus veins of wild‐type and Nur77 knockout mice. First, fresh blood was collected with a 0.5 ml collector containing coagulant and then placed at 4°C for 2 h. Finally, serum was collected by centrifugation, and the serum hsCRP and IL‐6 levels were determined using enzyme‐linked immunosorbent assay kits (Jianglaibio Co., Ltd).

### 2.4 MicroCT analysis

The trabecular bone volume of the femoral metaphysis was measured by MicroCT (Skyscan 1276; Bruker‐MicroCT). When scanning, the long axis of the tibia remained aligned with the scanning axis. Approximately 110 distal femur sections were examined at a resolution of 18 μm, and a continuous cross‐sectional image of the entire metaphyseal area was acquired using 88 keV and 309 μA Al_2_O_3_ and copper‐aluminum filters. Skyscan CTAn v.1.1.7 software (Skyscan CTAn) was used for the analysis of trabecular bone parameters, including the trabecular bone volume fraction bone volume/total volume (BV/TV), trabecular thickness (Tb. Th), trabecular number (Tb. N), trabecular separation (Tb. Sp), trabecular pattern factor (Tb. Pf), structure model index (SMI) and connectivity density (Conn. Dn).

### 2.5 Histomorphometric analysis

For histomorphometric analysis, femur bones were decalcified in 14% EDTA for 3 weeks after fixation overnight in 4% formalin until the bone was flexible. Then, they were dehydrated, cleared, waxed and embedded in paraffin. The femurs were subjected to serial paraffin sectioning (2 μm), and multinucleated osteoclasts (TRAP cells) were stained with tartrate‐resistant acid phosphatase (TRAP) according to the manufacturer's instructions (Servicebio). Haematoxylin and eosin staining was performed to observe trabecular bones, and the measurement range included the 3 mm secondary spongiosa, which began at the standard point below the growth plate at the end of the primary spongiosa of the femur. Three sections per mouse and five mice from each group were subjected to histomorphometric analysis.

### 2.6 Cell culture

RAW264.7 cells, a monocyte line, can be differentiated into several cell types; for example, they can be differentiated into osteoclasts by culturing in medium containing RANKL and M‐CSF.^28^ The RAW264.7 cells in our study were gifted by Professor Liu Cao (Institute of Health Sciences, China Medical University) and cultured in complete medium (DMEM, Hyclone; 14% FBS, Clark; 1% penicillin/streptomycin) at 37°C and 5% CO2. When the cells in a 10 cm culture dish reached nearly 80% confluence, they were passaged by trypsinization. A lentivirus was used to silence and overexpress Nur77 in RAW264.7 cells, and siRNAs were used to silence IκB‐α and IKK‐β. shRNA and siRNA oligonucleotide sequences are shown in Table S2.

### 2.7 Osteoclast differentiation and TRAP staining

The cells were seeded into a 24‐well plate at a density of 2 × 10^4^/well. After 24 h, the medium was replaced with DMEM containing 10% FBS, M‐CSF (50 ng/ml) and RANKL (50 ng/ml) for 5 days of induction (changing the medium every other day).^28^ Cells in the PDTC (inhibits NF‐κB activation by enhancing its binding to IκB‐α) treatment group were cultured in DMEM containing 10% FBS, 50 µM PDTC, 50 ng/ml M‐CSF and RANKL. Multinucleated (>3 nuclei) TRAP^+^ cells were defined as mature osteoclasts on day 5,^28^ and 5 fields of view were randomly selected under the microscope at a 10× objective to quantify the osteoclasts. Details of biomodulators are shown in Table S4.

### 2.8 Western blot

The details of the Western blot protocol have been described previously.^29^ Briefly, to obtain protein lysate, approximately 35 mg of femur or cells from the well of a 6‐well plate was placed in 200 μl of radioimmunoprecipitation assay lysis buffer. An enhanced chemiluminescence substrate (34580, Thermo Scientific) was used to evaluate the protein expression levels, and a Tanon ChemiDoc MP Imager was used to capture images. ImageJ software was used to quantify the densities of bands in the images, and a loading control (β‐actin) was used to normalize the protein level. Primary antibodies against TNF‐α, IL‐6, IκB‐α, IKK‐β, p‐IKK‐β, p‐NF‐kB, NF‐κB, β‐actin and Nur77 were used at a 1:1000 dilution. Sources and catalogs of antibodies are shown in Table S3.

### 2.9 Quantitative real‐time PCR

Total RNA was isolated from femurs using TRIzol reagent according to the manufacturer's instructions (Life Technologies). An iScript cDNA Synthesis Kit (BIO‐RAD, USA) was used to synthesize cDNA from 700 ng of total RNA, and Power Up SYBR Green master mix (Applied Biosystems, USA) and an LC480 II (Roche) qPCR instrument were used for real‐time quantitative PCR (qPCR). The qPCR results were calculated by the 2^−ΔΔCt^ method. Every sample was analysed in triplicate, and all experiments were repeated at least three times. The primer sequences used in our research are shown in Table S1.

### 2.10 Study population

To avoid the cold period, this research was conducted in Shenyang, Northeast China, from April to October 2018, and 6514 elderly residents were recruited. The exclusion criteria were as follows: participants who had musculoskeletal diseases, had malignant tumours, were under the age of 60, and had taken medications that affected bone or hsCRP metabolism, such as vitamin D, in the past year or for more than six months. Patients without complete information, such as the T‐score of the heel bone and frequency of physical activity, were also excluded. Finally, 4010 eligible subjects were included in this study, which was approved by the First Affiliated Hospital's Ethics Committee of China Medical University (Shenyang, China, ethics approval project identification code: AF‐SOP‐07‐1.0‐01). All investigations were conducted in accordance with the Declaration of Helsinki. All subjects signed an informed consent form, and the original data of this study are registered at the Chinese Clinical Trial Center (Registration Number: ChiCTR‐ERC‐17011100).

Standardized questionnaires were used to collect general information about the participants, including their gender, age, education status, medication, smoking and drinking history, form and frequency of physical activity, and were administered during face‐to‐face interviews. Sports activities mainly included jogging, walking, brisk walking, square dancing and Tai Chi. The frequency of exercise was classified as inactivity, 1–2 times/month, 1–2 times/week, 3–4 times/week or ≥5 times/week.

The height, weight, heel BMD and body muscle mass of each subject were measured by physical examination in accordance with previous studies.^29^, ^30^ Briefly, upon measurement, the subjects were required to wear light clothes and to take off their shoes; their height and weight were then measured accurately to 0.01 cm and 0.01 kg, respectively. A body fat metre (HBF‐701, Omron) was used to measure body muscle mass, and an ultrasonic bone densitometer (Hologic Sahara ultrasound bone density densitometer, software: version 3.1, American Hologic Corporation) was used to measure the heel BMD.^30^ Osteoporosis in this study was diagnosed based on a T‐score ≤−2.5 SD according to criteria recommended by the World Health Organization (WHO).

### 2.11 Serum parameters

Fasting blood samples were collected from individuals in the morning and stored at −80°C after centrifugation to reduce degradation. The latex‐enhanced immunoturbidimetric method was used to detect the serum hsCRP level. The clinical laboratory technicians were professionally trained, and all laboratory evaluations were performed in accordance with the standard operating procedures of hospital laboratories.

### 2.12 Statistical analysis

Statistical analyses were performed with SPSS v19.0 (Chicago, IL, USA). Differences in animal data were determined by Student's *t*‐test or one‐way ANOVA, and the Bonferroni method was used for post hoc comparisons. All results in the figures are presented as boxplots with median and interquartile ranges. Because the serum hsCRP concentrations in the participants demonstrated a positively skewed distributed, we created two level categories based on the median level (0.95 mg/L) and analysed the results with the Wilcoxon rank‐sum test. The mean ± standard deviation (SD) or frequency (%) was used for descriptive analyses of continuous and categorical variables, the differences in which were determined by Student's *t*‐test or the χ^2^ test. The relationship between the serum hsCRP level and osteoporosis was analysed by two separate multiple logistic regression models. A two‐tailed α level of 0.05 represented statistical significance.

## 3 RESULTS

### 3.1 Nur77 deletion accelerates bone loss and enhances osteoclast differentiation in mice

**FIGURE 1:**
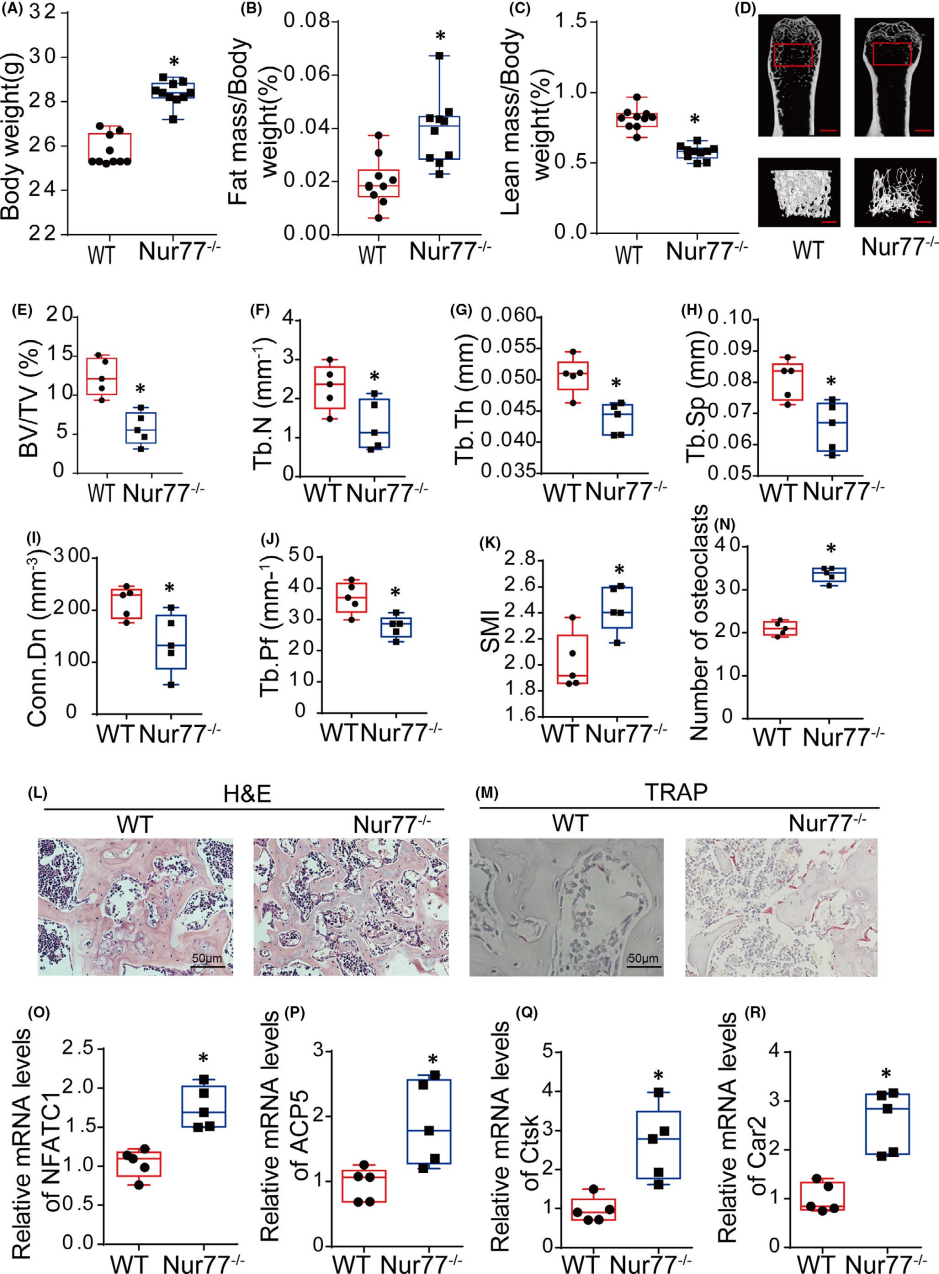
Nur77 deletion facilitates bone loss and osteoclastogenesis. (A‐C) Body weights and body compositions of the mice (*n* = 10). (D‐K) A three‐dimensional image of the femoral metaphysis trabecular bone (bottom), a two‐dimensional image of the entire distal femur trabecular bone (top) and quantification of the trabecular bone volume and architecture (*n* = 5), scale bar =0.3 mm. BV/TV, bone volume/tissue volume ratio; Tb. N, trabecular number; Tb. Th, trabecular thickness; Tb. Sp, trabecular separation; Conn. Dn, connectivity density; Tb. Pf, trabecular pattern factor; SMI, structure model index. (L‐M) Histomorphometric analysis by H&E staining (L) and TRAP staining (M). (N) Numbers of osteoclasts in Nur77 knockout and wild‐type littermate controls. Multinucleated TRAP+ (red) cells were identified as mature osteoclasts. (O‐R) The mRNA expression levels of osteoclast differentiation‐related factors, including NFATC1, ACP5, Ctsk and Car2, in the femurs of Nur77 knockout and wild‐type littermate controls (*n* = 5). Animal experiments were conducted using 8‐month‐old male mice. All data are shown as boxplots with medians and interquartile ranges and were statistically analysed by Student's *t*‐test. *p < 0.05 or *p* < 0.01 or *p* < 0.001

First, body complication analysis revealed that Nur77 knockout mice were fatter than wild‐type mice. As shown in Figure 1A, the body weight of Nur77 knockout mice was increased significantly compared with that of wild‐type mice (*p* < 0.001), which was reflected mainly in body fat (*p* < 0.001; Figure 1B), while the lean mass of knockout mice was decreased (*p* < 0.001; Figure 1C).

To determine whether Nur77 physiologically regulates bone growth, microCT was used to assess the *in vivo* skeletal phenotype of Nur77 knockout mice. Compared to their WT male littermate controls, the male Nur77 knockout mice had a lower bone mass (Figure 1D), manifested as a 53.43% lower BV/TV (*p* < 0.001; Figure 1E), 42.56% lower Tb. N (*p* < 0.05; Figure 1F), 13.69% lower Tb. Th (*p* < 0.05; Figure 1G) and 18.49% higher Tb. Sp (*p* < 0.05; Figure 1H). As a result, Conn. Dn was decreased by 36.26% (*p* < 0.05; Figure 1I), Tb. Pf was increased by 25.22% (*p* < 0.01; Figure 1J), and the SMI was increased by 20.56% (*p* < 0.05; Figure 1K); the 3D structure was quantified based on the relative amounts of plates (SMI = 0, strong bone) and rods (SMI = 3, fragile bone). In addition, histomorphometrical analysis of the femurs showed that the bone trabeculae after Nur77 knockout were rod‐shaped, with sparse connections and obvious fractures (Figure 1L), and the osteoclast number was significantly increased by 60.0% compared with that of WT mice (Figure 1M,N; *p* < 0.001).

Consistent with these observations, we also observed enhanced osteoclast differentiation in Nur77 knockout mice, as determined by the higher mRNA expression levels of osteoclast differentiation markers, including the master osteoclastogenic transcription factor NFATC1 (*p* < 0.01; Figure 1O), acid phosphatase 5, tartrate‐resistant (ACP5, also known as tartrate‐resistant acid phosphatase (Trap; *p* < 0.05; Figure 1P), cathepsin K (Ctsk; *p* < 0.05; Figure 1Q) and carbonic anhydrase 2 (Car2; Figure 1R). The microCT results did not reveal cortical damage after Nur77 knockout (data not shown).

These results suggest that Nur77 suppresses osteoclastogenesis during bone remodelling and that deletion of Nur77 reduces the bone mass primarily by increasing osteoclast differentiation.

### 3.2 Nur77 deletion improves serum inflammation levels and activates the NF‐κB signalling pathway in femur bone

Osteoclast differentiation can clearly be promoted by inflammatory cytokines, and Nur77 is a pivotal anti‐inflammatory regulator of numerous diseases. Therefore, we next investigated whether the increased number of osteoclasts in Nur77 knockout mice was related to increased inflammation.

**FIGURE 2:**
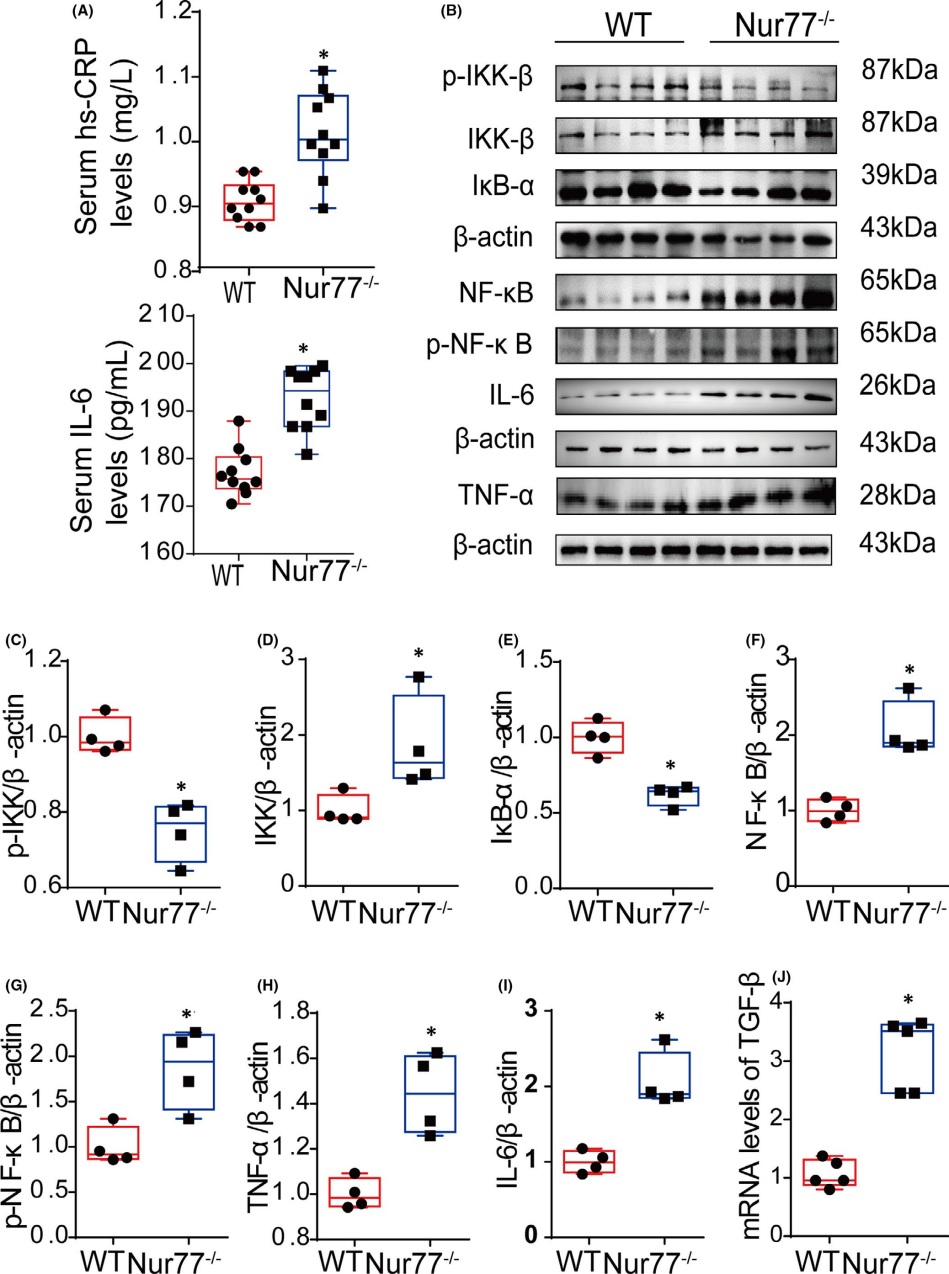
Nur77 deletion activates the NF‐κB signalling pathway in the femur. (A) The serum levels of hsCRP and IL‐6 in the mice (*n* = 10). (B‐I) The protein expression levels of P‐IKK‐β, IKK‐β, IκB‐α, NF‐κB, p‐NF‐κB, TNF‐α and IL‐6 in Nur77 knockout and wild‐type littermate controls. (J) The mRNA expression levels of TGF‐β in the mouse femurs. All data are presented as boxplots with medians and interquartile ranges and were statistically analysed by Student's *t*‐test. *p < 0.05 or *p* < 0.01 or *p* < 0.001

As shown in Figure 2A, the serum levels of hsCRP and IL‐6 were improved significantly in Nur77 knockout mice as determined by the ELISA (*p* < 0.001); coincidentally, many studies have reported that higher serum hsCRP and IL‐6 levels promote bone loss.^10^ In addition, in Nur77 knockout mice, the serum levels of LPS, TNF‐α and IL‐1β were previously shown to be increased significantly.^31^ This result mainly showed that the level of inflammation was increased in Nur77 knockout mice. To further clarify whether the increased number of osteoclasts in Nur77 knockout mice was associated with more inflammation, we next measured the levels of inflammation in mouse femurs.

Western blot revealed that in the femurs of Nur77 knockout mice, the protein expression levels of inflammatory factors that promote osteoclast production were significantly increased (Figure 2B‐J). The level of IKK‐β, which promotes NF‐kB activation, was increased (Figure 2D; *p* < 0.05); however, the level of its degraded form p‐IKK‐β was significantly reduced (Figure 2C; *p* < 0.01), and the expression of IKB‐α, which inhibits the translocation of NF‐κB into the nucleus, was significantly reduced (Figure 2E; *p* < 0.001). These factors increase the levels of NF‐kB (Figure 2F; *p* < 0.01), p‐NF‐kB (Figure 2G; *p* < 0.05) and the downstream signalling factors TNF‐α (Figure 2H; *p* < 0.01) and IL‐6 (Figure 2I; *p* < 0.01). Moreover, the mRNA level of TGF‐β was significantly increased in the femurs of Nur77 knockout mice as determined by real‐time PCR, which promoted osteoclast differentiation (Figure 2J; *p* < 0.001).

These results suggest that increased osteoclast differentiation in Nur77 knockout mice is related to the failure to suppress the NF‐kB signalling pathway.

### 3.3 Knockdown of Nur77 accelerates the differentiation of RAW264.7 cells into osteoclasts

**FIGURE 3:**
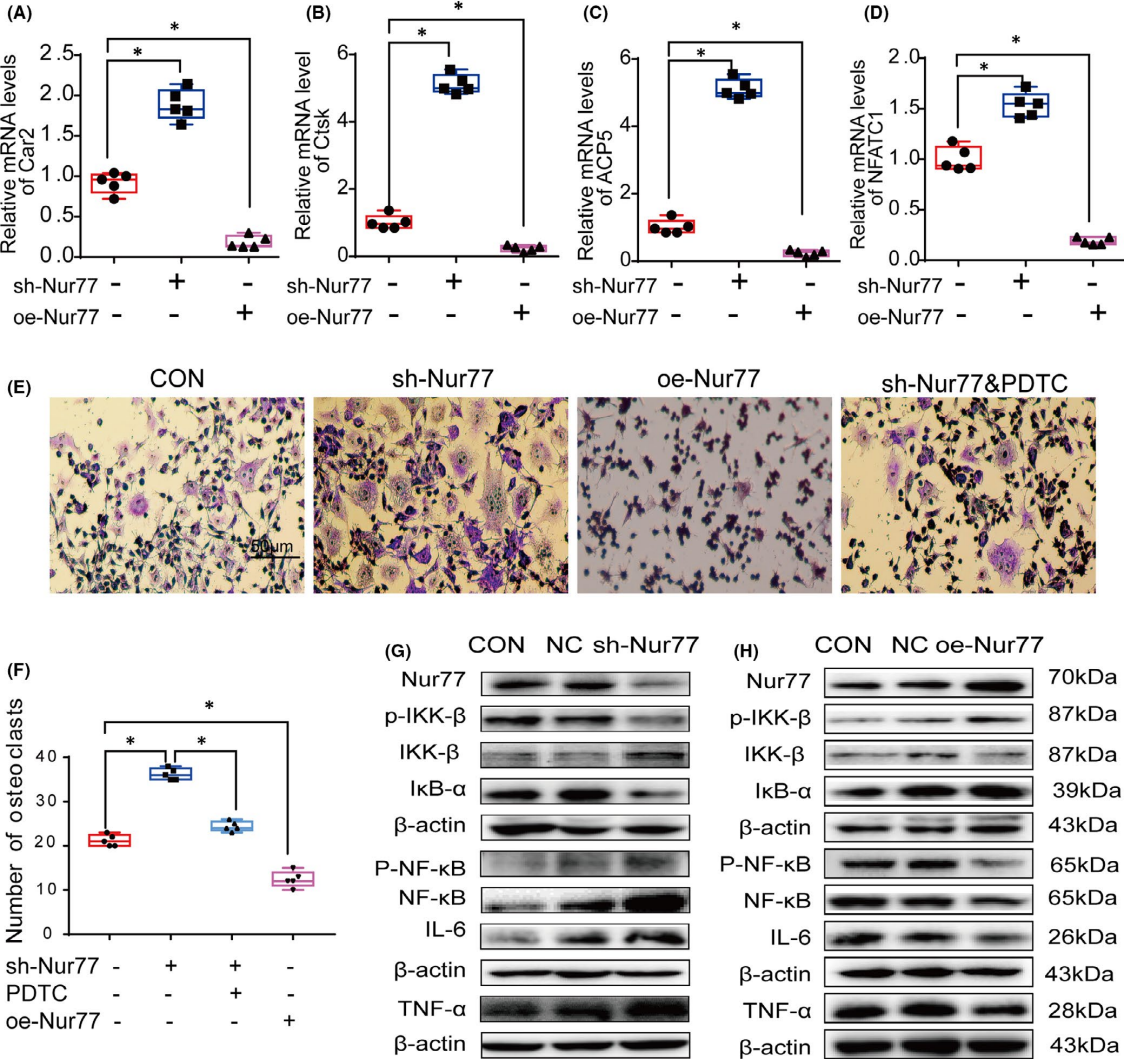
Nur77 suppresses the NF‐κB signalling pathway to inhibit osteoclast differentiation. (A‐D) Silencing Nur77 increased the expression of osteoclast differentiation factors, while overexpression of Nur77 had the opposite effect; the mRNA expression levels of Car2 (A), Ctsk (B), ACP5, (C) and NFATC1 (D) were analysed. (E‐F) Deletion of Nur77 increased the potential for RAW264.7 cells to differentiate into osteoclasts; however, PDTC (an inhibitor of NF‐κB) reversed this phenomenon. (E) TRAP staining image of osteoclast differentiation cultures. Multinucleated TRAP+ (red) cells were identified as mature osteoclasts on day 5. (F) Osteoclast numbers. (G) Silencing Nur77 activated the NF‐κB signalling pathway and increased the levels of TNF‐α and IL‐6 in RAW264.7 cells, while (H) overexpressing Nur77 inhibited the NF‐κB signalling pathway and decreased the TNF‐α and IL‐6 expression in RAW264.7 cells. All data are presented as boxplots with median and interquartile range and were statistically assessed by one‐way analysis followed by the Bonferroni test. *p < 0.001

**FIGURE 4:**
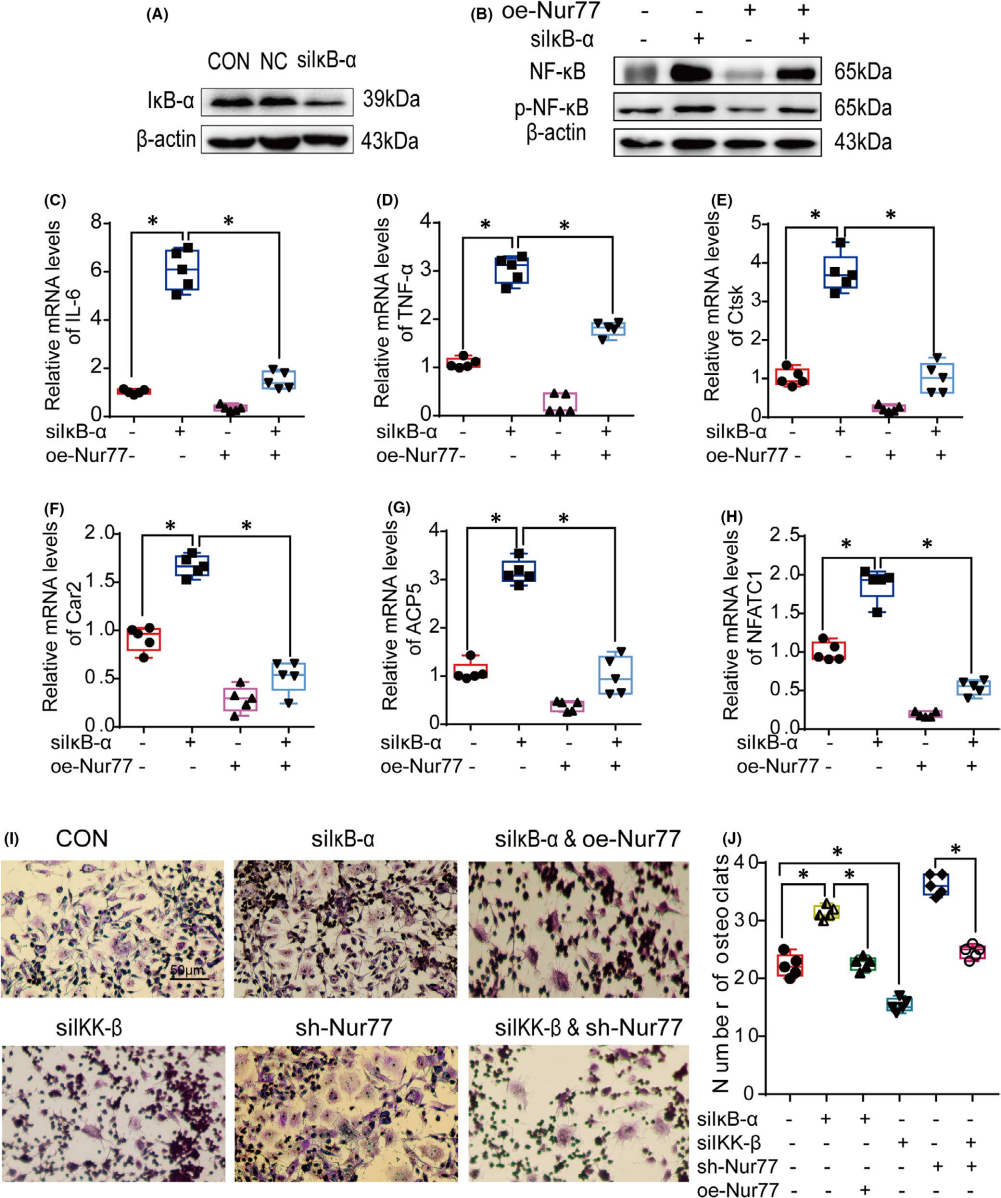
Nur77 inhibits the activation of NF‐κB by upregulating IκB‐α expression during osteoclast differentiation. (A) The protein levels of IκB‐α. (B) The protein levels of NF‐κB and p‐NF‐κB. (C‐H) The mRNA levels of IL‐6, TNF‐α, Ctsk, Car2, ACP5 and NFATC1. (I) TRAP staining image of osteoclast differentiation cultures. Multinucleated TRAP+ (red) cells were identified as mature osteoclasts on day 5. (J). Osteoclast numbers. All data are presented as boxplots with median and interquartile range and were statistically assessed by one‐way analysis followed by the Bonferroni test. *p* < 0.001

An early study demonstrated that RAW264.7 cells can differentiate into osteoclasts after induction by RANKL and M‐CSF.^28^ To validate our *in vivo* results and further explore the role of Nur77 in osteoclast differentiation, we examined the differentiation of RAW264.7 cells into osteoclasts after the gain and loss of Nur77 function. RAW264.7 cells with stable Nur77 overexpression and Nur77 knockdown were established by lentivirus infection, and the efficiencies were assessed by western blotting (Figure 3G,H). As expected, the deletion of Nur77 increased the mRNA expression of osteoclast differentiation markers (Figure 3A‐D), including Car2, Ctsk, ACP5 and NFATC1 (all of the *p* < 0.001), and the number of mature osteoclasts was increased compared with that in the control group (Figure 4E,F; *p* < 0.001). In contrast, overexpressing Nur77 suppressed the mRNA expression of osteoclast differentiation markers (Figure 3A‐D; all of the *p* < 0.001) and resulted in fewer osteoclasts (Figure 3E,F; *p* < 0.001).

These results indicate that Nur77 protects bone tissue by inhibiting the differentiation of osteoclasts.

### 3.4 Nur77 suppresses the NF‐κB signalling pathway to inhibit osteoclast differentiation

*In vivo* experiments showed that the NF‐κB signalling pathway was activated in the femurs of Nur77 knockout mice. To explore the potential mechanism by which Nur77 inhibits osteoclast differentiation by inhibiting the NF‐κB signalling pathway, we detected the relative protein expression levels of NF‐κB signalling components in RAW264.7 cells lacking Nur77. The protein levels of IKK‐β and NF‐κB were increased (Figure 3G), while those of IκB‐α and p‐IKK‐β were decreased significantly (Figure 3G); moreover, the expression of p‐NF‐κB, IL‐6 and TNF‐α was increased (Figure 3G). However, in Nur77‐overexpressing RAW264.7 cells, the levels of IKK‐β and NF‐κB were downregulated (Figure 3H), while those of IκB‐α and p‐IKK‐β were increased (Figure 3H), which resulted in decrease levels of p‐NF‐κB, IL‐6 and TNF‐α (Figure 3H).

To further confirm our hypothesis, we treated Nur77‐silenced RAW264.7 cells with PDTC, an inhibitor of NF‐κB. TRAP staining revealed that PDTC significantly reduce the number of mature osteoclasts (Figure 3E,F; *p* < 0.001).

The above results strongly suggest that Nur77 inhibits the differentiation of osteoclasts by suppressing the activation of the NF‐kB signalling pathway.

### 3.5 Nur77 inhibits the activation of the NF‐κB signalling pathway by upregulating IκB‐α expression and reducing IKK‐β expression during osteoclastogenesis

The NF‐κB signalling pathway is regulated by IκB‐α and IKK‐β. As shown above, the *in vivo* experiments indicated that Nur77 deletion downregulated IκB‐α expression while upregulating that of IKK‐β in the mouse femur. To clarify whether the Nur77‐mediated regulation of inflammation depends on IκB‐α and IKK‐β, we silenced IκB‐α and IKK‐β in RAW264.7 cells using siRNAs (Figure 4A,B).

Western blot analysis demonstrated that siIκB‐α treatment (Figure 4A) significantly increased the protein levels of NF‐κB and p‐NF‐κB (Figure 4B) and the mRNA levels of IL‐6 and TNF‐α (Figure 4C,D) in RAW264.7 cells (all *p* < 0.001). Moreover, the levels of the osteoclast differentiation‐related factors Ctsk, Car2, ACP5 and NFATC1 were increased significantly (Figure 4E‐H; all *p* < 0.001). Consistent with these changes, the number of osteoclasts was increased in siIκB‐α‐treated cells (Figure 4I,J; *p* < 0.001), while Nur77 overexpression mitigated this increase.

**FIGURE 5:**
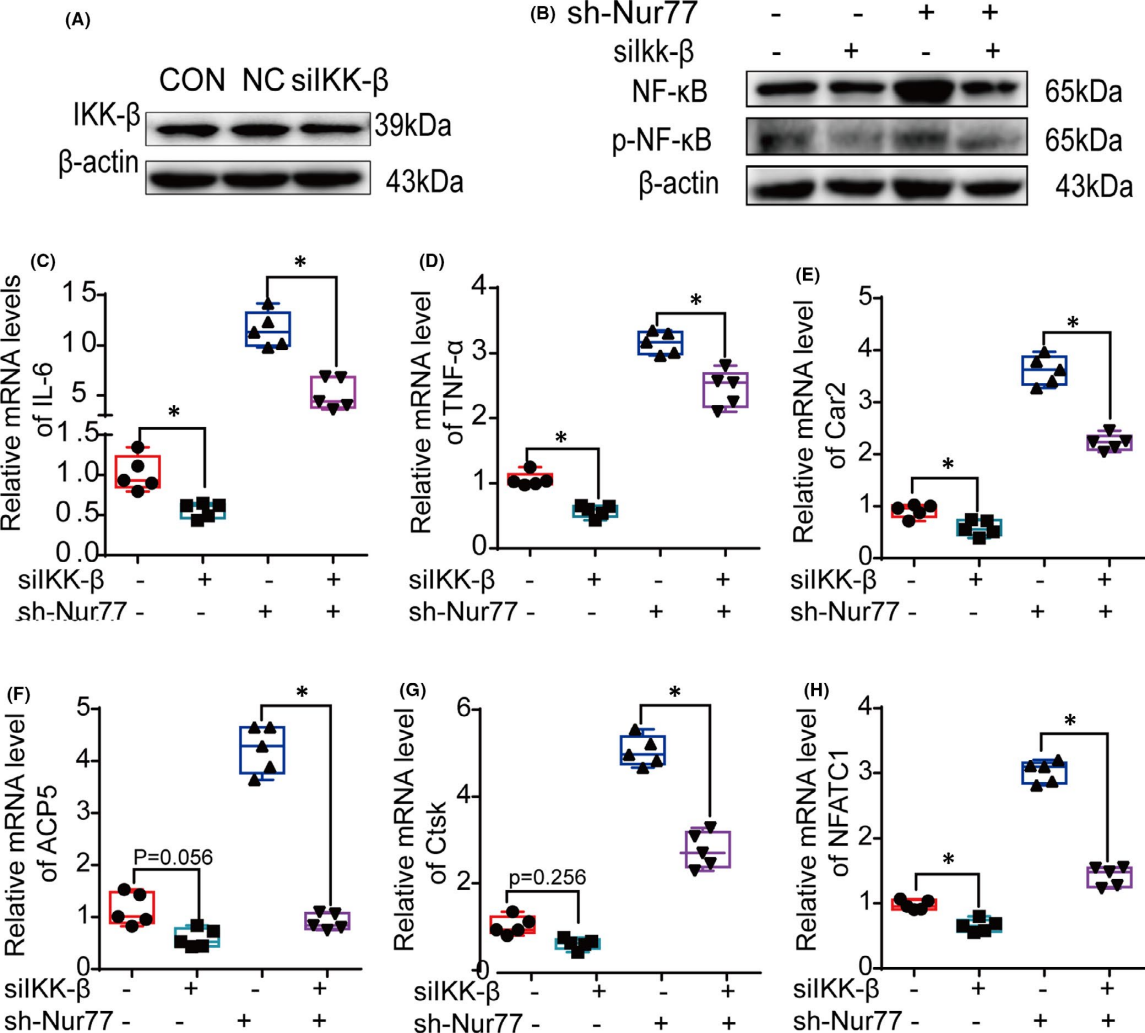
Nur77 inhibits the activation of NF‐κB by downregulating the expression of IKK‐β during osteoclast differentiation. (A) The protein levels of IKK‐β. (B) The protein levels of NF‐κB and p‐NF‐κB. (C‐H) The mRNA levels of IL‐6, TNF‐α, Car2, ACP5, Ctsk and NFATC1. All data are presented as boxplots with median and interquartile range and were statistically assessed by one‐way analysis followed by the Bonferroni test. *p* < 0.001

In addition, siIKK‐β treatment (Figure 5A) significantly reduced the protein levels of NF‐κB and p‐NF‐κB (Figure 5B), which thereby decreased the levels of IL‐6 and TNF‐a (Figure 5C,D; all *p* < 0.001), and the mRNA levels of Car2 and NFATC1 were downregulated (Figure 4E,H; all *p* < 0.001). Moreover, siIKK‐β alleviated the increase in inflammation and osteoclastogenesis after Nur77 deletion, which was mainly manifested by not only the decreased expression of NF‐κB and p‐NF‐κB (Figure 5B) and the reduced mRNA expression of IL‐6, TNF‐α and osteoclast differentiation‐related factors (Figures 5C‐H; all of the *p* < 0.001) but also the lower osteoclast numbers in the siIKK‐β and shNur77 groups (Figure 4I,J; *p* < 0.001).

This evidence strongly supports the notion that Nur77 regulates both IκB‐α and IKK‐β during osteoclastogenesis.

### 3.6 Elderly individuals with high levels of inflammation are highly susceptible to osteoporosis

Although many studies have shown that higher serum hsCRP levels are related to osteoporosis, some authors do not believe that these two factors are not related,^32^, ^33^ which is contrary to our results. To make our conclusion more credible, we assessed the relationship between the serum level of hsCRP and osteoporosis in an elderly population.

As shown in Table 1, the participants were grouped according to their serum hsCRP levels. The frequency distributions of gender, education level and physical activity differed among the groups (all *p* < 0.001), and women with low education levels and physical activity were associated with higher levels of serum hsCRP. The mean values of age, weight, height, BMI, body muscle, BMD and T‐score differed among the groups with varying serum hsCRP levels (all *p* < 0.05). Compared with those of participants with serum hsCRP levels below the median, the BMI of those with serum hsCRP levels above the median was 6.0% higher, while their body muscle mass was 4.3% lower, their BMD was 7.1% lower, and their T‐score was 12.8% lower.

**TABLE 1:** Descriptive characteristics of the study population stratified by serum hsCRP level

Variables	hsCRP below median (<0.95 mg/L, *n* = 2003)	hsCRP above median (≥ 0.95 mg/L, *n* = 2007)	*p*
Age, years	68.42 ± 6.16	69.29 ± 6.70	<0.001
Gender			0.001
Male, *n* (%)	882 (53.1)	778 (46.9)	
Female, *n* (%)	1121 (47.7)	1229 (52.3)	
Education level			<0.001
Illiteracy or Elementary school	305 (41.9)	423 (58.1)	
Junior high school or senior high school	1346 (50.6)	1314 (49.4)	
College	352 (56.6)	270 (43.4)	
Weight, kg	63.33 ± 23.79	65.63 ± 21.21	0.01
Height, cm	161.02 ± 10.23	160.14 ± 9.67	0.024
BMI, kg/m^2^	24.06 ± 3.07	25.50 ± 3.59	<0.001
Smoking status			0.114
Yes, *n* (%)	274 (53.2)	241 (46.8)	
No, *n* (%)	1729 (49.5)	1766 (50.5)	
Drinking status			0.947
Yes, *n* (%)	622 (51.2)	594 (48.8)	
No, *n* (%)	1381 (49.4)	1413 (50.6)	
Physical activity			
Active, *n* (%)	1412 (52.2)	771 (47.8)	<0.001
Moderate, *n* (%)	223 (47.1)	250 (52.9)	
Inactive, *n* (%)	368 (44.2)	464 (55.8)	
Body muscle mass, %	25.54 ± 3.71	24.44 ± 3.46	<0.001
BMD, g/cm^2^	0.411 ± 0.158	0.381 ± 0.165	<0.001
T‐score	−1.56 ± 1.43	−1.76 ± 1.49	<0.001

As shown in Table 2, differences in the frequency distributions of the basic characteristics of the subjects, such as their gender, education level, drinking status and physical activity, were statistically significant (all *p* < 0.05) after grouping according to the T value. Compared with the control group, women who had low education levels, low activity and drank alcohol were more likely to suffer from osteoporosis. The average values for age, weight, body muscle mass and BMD also differed significantly between the control group and the osteoporosis group (all *p* < 0.05). The body muscle mass of the osteoporosis group was 1.3% lower than that of the control group, while the BMD was 50.95% lower. The median hsCRP concentration in subjects with osteoporosis was 1.17 mg/L, with 25th and 75th percentiles of 0.60 and 2.90 mg/L, respectively. In the control group, the median hsCRP concentration was 0.89 mg/L (quartile range = (0.15 mg/L, 1.77 mg/L)).

**TABLE 2:** Descriptive characteristics of the study population stratified by T‐score

Variables	Control (*n* = 2807)	Osteoporosis (*n* = 1203)	*p*
Age, years	68.4 ± 6.2	69.9 ± 6.9	<0.001
Gender			0.008
Male, *n* (%)	1200 (72.3)	460 (27.7)	
Female, *n* (%)	1607 (68.4)	743 (31.6)	
Education level			<0.001
Illiteracy or Elementary school	460 (63.2)	268 (36.8)	
Junior high school or senior high school	1892 (71.1)	768 (28.9)	
College	455(73.2)	167 (26.8)	
Weight, kg	64.72 ± 17.80	63.36 ± 11.64	0.019
Height, cm	160.57 ± 9.74	160.60 ± 10.55	0.948
BMI, kg/m^2^	24.84 ± 3.21	24.64 ± 3.84	0.086
Smoking status			0.797
Yes, *n* (%)	2444 (69.9)	1051 (30.1)	
No, *n* (%)	363 (70.5)	152 (29.5)	
Drinking status			0.045
Yes, *n* (%)	1929 (69.0)	865 (31.0)	
No, *n* (%)	878 (72.2)	338 (27.8)	
Physical activity			0.003
Active, *n* (%)	1937 (71.6)	768 (28.4)	
Moderate, *n* (%)	307(64.9)	166(35.1)	
Inactive, *n* (%)	563(67.7)	269(32.3)	
Body muscle mass, %	25.09 ± 3.70	24.76 ± 3.45	0.008
BMD, g/cm^2^	0.474 ± 0.133	0.233 ± 0.064	<0.001
hsCRP (mg/L) (median, interquartile range)	0.89(0.51–1.77)	1.17(0.60–2.90)	<0.001

To further explore the relationship between hsCRP and osteoporosis, we conducted logistic regression analysis, and the results are shown in Table 3. Serum hsCRP was associated with osteoporosis in the unadjusted model (OR = 1.91, 95% CI = (1.66–2.20), *p* < 0.001). In model 1, after adjusting for age and sex, the result was similar to that of the unadjusted model. We then adjusted for age, sex, BMI, education level, body muscle mass, smoking status, drinking status and physical activity in model 2, and the association between the serum hsCRP level and osteoporosis remained statistically significant (OR = 1.76, 95% CI: 1.51–2.06, *p* < 0.001).

**TABLE 3:** Multiple logistic regression analyses to determine the odds ratios for osteoporosis according to serum hsCRP level in participants

Model	OR	95%CI	*p*
unadjusted	1.91	1.66–2.20	<0.001
Model 1	1.84	1.60–2.13	<0.001
model 2	1.76	1.51–2.06	<0.001

Model 1 adjusted for age, gender.

Model 2 adjusted for age, gender, BMI, Education level, body muscle mass, Smoking status, drinking status, physical activity.

These results suggested that elderly individuals with high serum hsCRP levels were more likely to suffer from osteoporosis than those with low levels, which was consistent with the *in vivo* experimental results.

## 4 DISCUSSION

The main findings of this study were that by inhibiting the NF‐κB signalling pathway, Nur77 suppresses osteoclast differentiation, which is mainly related to the upregulation of IκB‐α and downregulation of IKK‐β.

Previous studies have found that Nur77 inhibits the translocation of NF‐κB to the nucleus by binding to P65 during the inflammatory response induced by LPS, thereby suppressing the inflammatory response.^34^ Moreover, Nur77 exerts anti‐inflammatory and antioxidant stress effects by inhibiting IκB‐α phosphorylation in a cell model of Parkinson's disease.^31^ In the present study, *in vivo* and *in vitro* functional studies revealed that the osteoprotective effect of Nur77 essentially involves the inhibition of osteoclast differentiation via the exertion of anti‐inflammatory activity. Two studies showed that Nur77 deletion resulted in osteoporosis, but the level of inflammation in the bones was not changed.^35^, ^36^ The experimental animals selected in these two previous studies were 4 or 5 months old; thus, these animals were younger than those in our study (8 months old), and the compensatory ability of the immune system was stronger. This may be related to their failure to find an anti‐inflammatory effect of Nur77 in bone. In addition, in our cross‐sectional study, older participants with higher serum hsCRP levels were more likely to suffer from osteoporosis, which was consistent with the *in vivo* experimental results. The relationship between Nur77 and osteoporosis was not studied directly in this population, potentially make the conclusions of animal studies more reliable. Thus, combining the *in vivo* and *in vitro* experimental results and the conclusions of the cross‐sectional study, our study strongly clarified that by suppressing the NF‐κB signalling pathway, Nur77 downregulates the expression of hsCRP, IL‐6 and TNF‐α, thereby effectively preventing osteoclast differentiation and osteoporosis.

NF‐κB plays a pivotal role in the inflammatory response. At rest, it is isolated in the cytoplasm by IκB‐α and IκB‐β^37^, ^38^; however, IκB‐α is degraded upon activation of the inflammatory response. *In vivo*, we found that the total expression of IκB‐α was significantly reduced in Nur77 knockout mice and that its ability to inhibit NF‐κB phosphorylation and translocation into the nucleus was weakened, similar to previous studies on endothelial cell activation and leukocyte adhesion.^39^ For further exploration, we silenced IκB‐α with siRNA in RAW264.7 cells and found that both the protein expression levels of NF‐κB and p‐NF‐κB and the mRNA levels of osteoclast differentiation‐related factors were increased significantly, while the number of mature osteoclasts formed by the differentiation of RAW264.7 cells was enhanced. Nevertheless, these effects were attenuated by overexpressing Nur77, and these results indicated that the potential of Nur77 to inhibit osteoclast differentiation is related to its promotion of IκB‐α. In addition, external stimuli phosphorylate and thus activate IKK‐β, which in turn induces the translocation of NF‐κB into the nucleus.^39^, ^40^, ^41^ We demonstrated that the protein expression levels of IKK‐β and p‐IKK‐β were increased in Nur77 knockout mice, consistent with previous research showing that Nur77 protected against myocardial ischaemic injury.^42^ For a more detailed investigation, we also silenced IKK‐β in RAW264.7 cells, which decreased the protein expression levels of NF‐κB and p‐NFκB in RAW264.7 cells. Furthermore, silencing IKK‐β alleviated the excessive inflammation caused by Nur77 knockout and inhibited its osteoclast differentiation potential, indicating that the potential of Nur77 to inhibit osteoclast differentiation is related to the downregulation of IKK‐β. Interestingly, we found that after silencing IKK‐β, only the NFATC1 mRNA levels of osteoclast differentiation‐related factors were reduced significantly. However, previous studies demonstrated that the expression of NFATC1 in osteoclast precursors is sufficient to induce osteoclast differentiation,^43^ and the reliability of the conclusion drawn herein is thus not weakened. Thus, we suggest that Nur77 inhibits the NF‐κB signalling pathway during osteoclast differentiation by both promoting the production of IκB‐α and decreasing the expression of IKK‐β, and these phenomena work together to inhibit the translocation of NF‐κB into the nucleus.

Overall, our study suggests that Nur77 protects against osteoporosis by restricting inflammation, thus preventing osteoclast differentiation. The findings of this study provide a new perspective and target for the prevention and treatment of osteoporosis. Because age is an essential factor leading to inflammatory response activation and closely related to osteoporosis, future studies need to determine whether osteoporosis caused by ageing is related to changes in Nur77 expression.

## CONFLICT OF INTEREST

The authors declare that they have no known competing financial interests or personal relationships that could have influenced the work reported in this paper.

## AUTHOR CONTRIBUTION

**Huanlian Tian:** Conceptualization (equal); Investigation (equal); Methodology (equal); Validation (equal); Visualization (equal); Writing – original draft (equal). **Feng Chen:** Conceptualization (equal); Investigation (equal); Project administration (equal). **YIngfang Wang:** Investigation (equal); Project administration (equal); Visualization (equal). **Yixuan Liu:** Methodology (equal); Project administration (equal); Writing – review & editing (equal). **Guojing Ma:** Investigation (equal); Methodology (equal). **Yuhong Zhao:** Methodology (equal); Resources (equal). **Yanan Ma:** Formal analysis (equal); Methodology (equal). **Tingting Tian:** Investigation (equal); Validation (equal). **Ruze Ma:** Investigation (equal); Software (equal). **Yang Yu:** Conceptualization (equal); Methodology (equal); Project administration (equal); Visualization (equal). **Difei Wang:** Conceptualization (equal); Funding acquisition (equal); Methodology (equal); Project administration (equal); Resources (equal); Supervision (equal); Writing – review & editing (equal).

## Supporting information

Table S1‐S4
