# Supplementary material for: Nur77 Prevents Osteoporosis by Inhibiting the NF‐κB Signalling Pathway and Osteoclast Differentiation
Source: J Cell Mol Med. 2022 Feb 19;26(8):2163–76. doi: 10.1111/jcmm.17238 (PMC8995449; doi:10.1111/jcmm.17238)
Supplement: Supplementary file 1 — Table S1‐S4 [file JCMM-26-2163-s001.docx]

Supplementary Table 1. Primers used for qRT-PCR

| **Gene Name** | **Forward Primer** | **Reverse Primer** |
| --- | --- | --- |
| IL-6 | CTTCCATCCAGTTGCCTTCT | CTCCGACTTGTGAAGTGGTATAG |
| TNF-α | CCAGCCGATGGGTTGTACCT | CAAATCGGCTGACGGTGTGG |
| TGF-β | GGGCTTTCGATTCAGCGCTC | TCATGGATGGTGCCCAGGTC |
| Car2 | CTCTCTGACCACTCCGCCTCTG | ATCTGCTCGCTGCTGACAGTAATG |
| Ctsk | GTTGGGCTTTCAGCTCTGCC | ATATAGCCGCCTCCACAGCC |
| ACP5 | CACTCCCACCCTGAGATTTG | CGGTTCTGGCGATCTCTTT |
| NFATC1 | CATCCTTGCCTGCCCTTGACTG | GACCTATCGCAGTTGAAGT |

Supplementary Table 2. shRNA and siRNA oligonucleotide sequences

| Gene Target | Forward Primer |
| --- | --- |
| sh-Con  sh-Nur77  siCon for IKK-β | CCGGTTCTCCGAACGTGTCACGTTTCAAGAGAACGTGACACGTTCGGAGAATTTTTTG  CGGCCTGGCATACCGATCTAAATTCAAGAGATTTAGATCGGTATGCCAGGTTTTTTG  UUCUCCGAACGUGUCACGUTT  UUCUCCGAACGUGUCACGUTT |
| SiIKK-β | GAGAGAAAAGAUUAAUACATT |
| siCon for IκB-α | UUCUCCGAACGUGUCACGUTT |
| siIκB-α | GUACAAAUAGAGUUUUAUUTT |

Supplementary Table 3. Antibodies used for Western blotting

| **Antibody** | **Source and Catalog #** | **Company** |
| --- | --- | --- |
| Anti-TNF-α | Rabbit, 11948 | CST |
| Anti-IL-6 | Rabbit, 12912 | CST |
| Anti-IқB-α | Rabbit, 4812 | CST |
| Anti-IKK-β | Rabbit, 2370 | CST |
| Anti-p-IKK-β | Rabbit, 2697 | CST |
| Anti-p-NF-kB | Rabbit, 3033 | CST |
| Anti-NF-κB | Rabbit, 8242 | CST |
| Anti-β-actin | Rabbit, 2118 | CST |
| Anti-Nur77 | Rabbit, Sc-166166 | Santa Cruz |

Supplementary Table 4. Biological Modulators

| **Modulator** | **Source and Catalog #** | **Solvent/Vehicle** | **Concentration** | |
| --- | --- | --- | --- | --- |
| RANKL | R&D Systems, 462-TEC-010 | 0.1%BSA | | 50 ng/ml |
| M-CSF | R&D Systems, 416-ML | 0.1%BSA | | 50 ng/ml |
| PDTC | Beyotime, S1809 | DMSO | | 50 μM |
